# Supplementary material for: A Comparative Investigation of the Surface Properties of Corn-Starch-Microfibrillated Cellulose Composite Films
Source: Materials (Basel). 2023 Apr 23;16(9):3320. doi: 10.3390/ma16093320 (PMC10179309; doi:10.3390/ma16093320)
Supplement: Supplementary file 1 [file materials-16-03320-s001.zip › materials-2343247-supplementary.pdf]

Supplementary Materials

A Comparative Investigation of the Surface Properties of Corn Starch-Microfibrillated Cellulose Composite Films

Zuzanna Żołek-Tryznowska [1,*], Ewa Bednarczyk [1], Mariusz Tryznowski [1] and Tomasz Kobiela [2]

[1] Faculty of Mechanical and Industrial Engineering, Warsaw University of Technology, Narbutta 85, 02-524, Warsaw, Poland
[2] Faculty of Chemistry, Warsaw University of Technology, Noakowskiego 3, Warsaw, 00-662, Poland
***** Correspondence: Zuzanna.Tryznowska@pw.edu.pl;

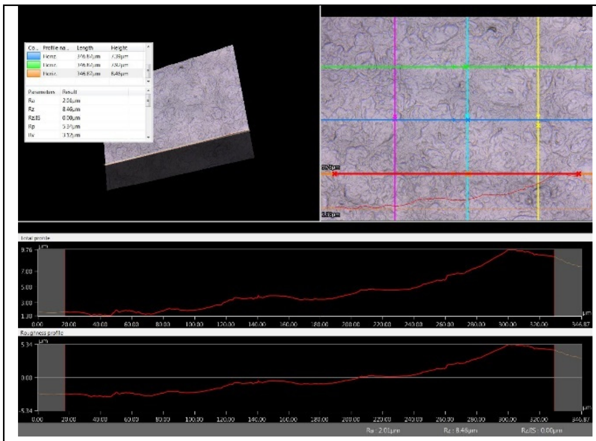

S_0

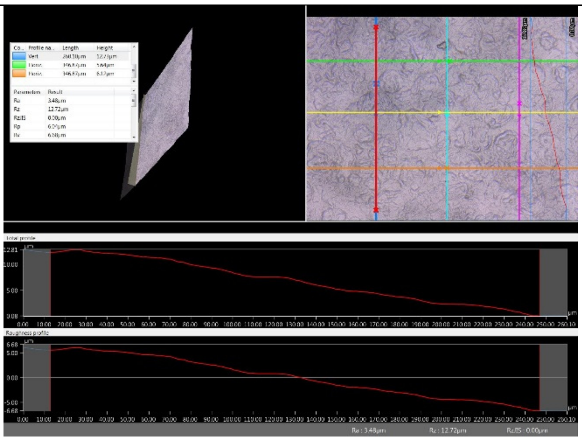

S_0.2

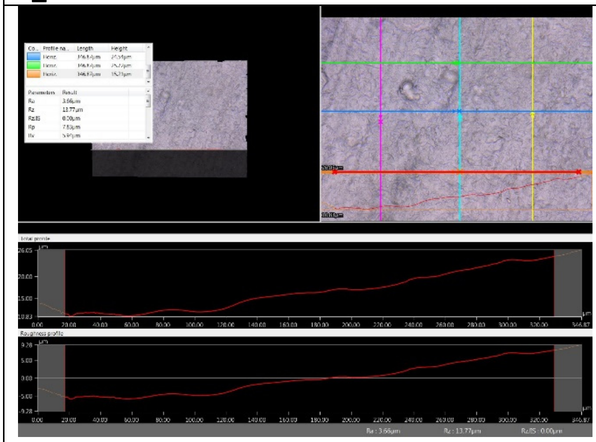

S_0.6

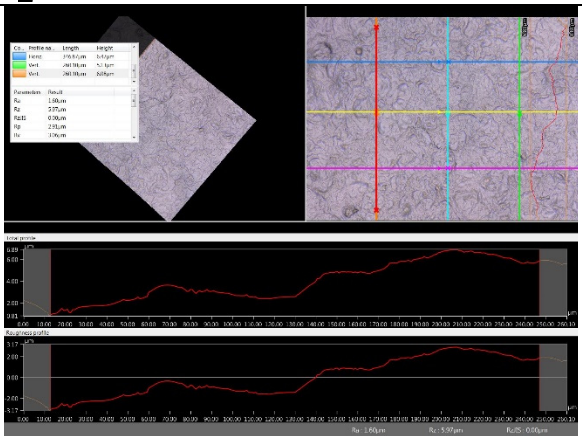

S_1

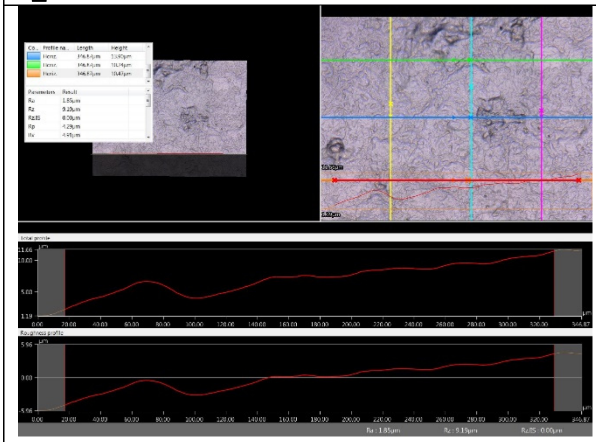

S_2

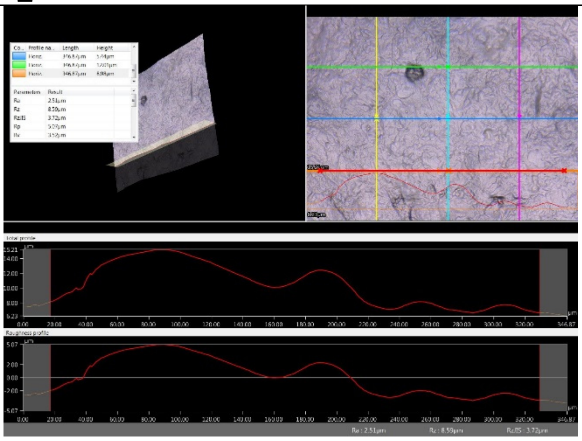

S_3

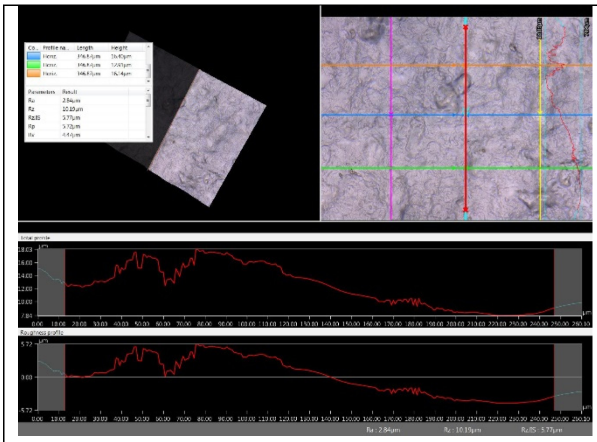 **S_4**	

Figure S1: Images of microscopic observation of developed film surface obtained with optical microscope.

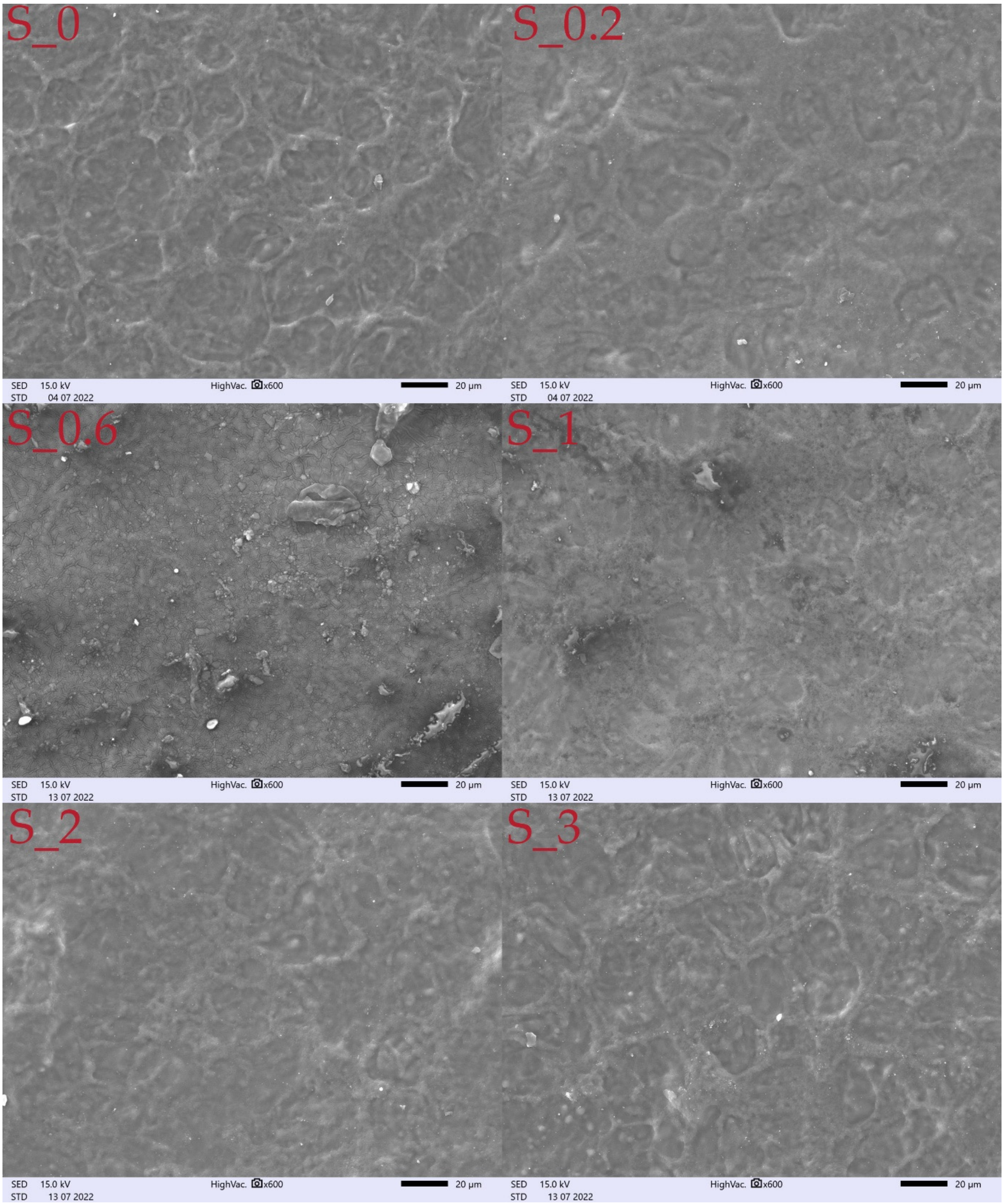

Figure S2: SEM **i**mages of surface at a magnification of 600x.
